# Reactions to an Online Demonstration of the Effect of Increased Fruit and Vegetable Consumption on Appearance: Survey Study

**DOI:** 10.2196/15726

**Published:** 2020-07-14

**Authors:** Patrick Cairns, Gozde Ozakinci, David Ian Perrett

**Affiliations:** 1 Perception Lab School of Psychology & Neuroscience University of St Andrews St Andrews United Kingdom; 2 School of Medicine University of St Andrews St Andrews United Kingdom

**Keywords:** diet, skin appearance, motivations, fruit and vegetables, carotenoid

## Abstract

**Background:**

Inadequate fruit and vegetable consumption causes a considerable disease burden and premature mortality. Despite public health promotion of a healthy diet, the average consumption is still below recommended levels. Fruit and vegetable consumption influences human skin color, increasing red/yellow/orange pigment in the skin. Given that this color is deemed attractive and healthy-looking, the appearance benefit may motivate to eat more fruit and vegetables. Such appearance motivation could be particularly useful in young individuals who currently eat the least fruit and vegetables.

**Objective:**

Our objectives were to assess how widely the impact of diet on skin color is known within the UK and to compare the strength of motivation to eat fruit and vegetables based on health and appearance benefits among different demographic groups.

**Methods:**

Four groups of UK residents (N=200 per group) were recruited through the Prolific online platform. Groups comprised younger (aged 18-24) and older adults (aged 40-60) of low and high self-reported socioeconomic status (1-5 and 6-10 on a 10-point rating scale). Facial images simulating the skin color associated with low and high fruit and vegetable diets were shown to participants. Questionnaires were used to assess (1) background knowledge of the health and skin color effects of dietary fruit and vegetables, (2) the specific motivational impact of the skin color illustration, and (3) the relative importance of motivation to consume fruit and vegetables arising from health and skin color appearance benefits.

**Results:**

We found that 61% (n=487) of all participants were unaware of the dietary–skin color association. We also found that 57% (n=457) of participants found the simple demonstration of the dietary impact on skin color positively motivating to eat more fruit and vegetables. The visual demonstration was equally motivating for participants of high and low self-reported socioeconomic status (*P*=.63) and different ethnic backgrounds (White N=453, Black N=182, Asian N=87, *P*=.22). Health benefits from a diet high in fruit and vegetables were regarded as more motivating than skin color appearance benefits. The appearance-changing benefits of a high fruit and vegetable diet (compared to the health benefits) were relatively more important for the younger participants (Mann-Whitney U=96,263, *P*<.001) and for women (N=489) than for men (N=310, U=83,763, *P*=.01).

**Conclusions:**

These findings indicate that the promotion of the skin color effects of diets high in fruit and vegetables could provide additional motivation for a healthier diet. Our study indicates the broad appeal of appearance benefits from dietary fruit and vegetable (across ethnicity and socioeconomic status) and particularly amongst young adults where an inadequate diet is most prevalent.

## Introduction

### Background

Inadequate fruit and vegetable consumption is estimated to lead to between 5.6 to 7.8 million premature deaths per year worldwide [1], chiefly through incidences of cardiovascular disease including coronary heart disease and stroke [1-3], diabetes and its complications [4,5] and several cancers [1,2,6]. Globally inadequate intake of fruit and vegetables is also responsible for the loss of up to 103 million years of healthy life due to disability [7]. The negative ramifications of these lifestyle-attributable diseases are widely felt. In addition to the consequences for personal wellbeing, poor population health contributes to an overburdening of healthcare systems and fiscal strain due to lost productivity [8].

Only 29% of adults in the UK [9] report eating the recommended 5 portions of fruit and vegetables per day. Fewer men (26%) than women (32%) meet the 5 a day guideline. Young people aged 16-24 were also less likely than other adults aged 45-60 to get their 5 a day (average 3.3 portions per day compared to 3.8). Figures are worse for children, with only 18% of children aged 5-15 eating 5 portions per day. The National Diet and Nutrition Survey showed that the proportion of adults (~30%) consuming the 5 a day recommendation has changed little over the last decade [10]. Residential area deprivation and lower socioeconomic status independently predict decreased fruit and vegetable consumption [11,12].

Health promotion efforts to increase fruit and vegetable consumption vary from individual approaches such as personal (or parental) advice and counseling to public health campaigns. Although much has been achieved in terms of understanding what psychological techniques may work or not to facilitate healthy eating [13], given the current state of inadequate consumption of fruit and vegetables, we are still in need of novel and innovative methods in addition to existing ones to encourage healthy eating.

### Skin Color Effects

Fruit and vegetable consumption influences human skin color, increasing the presence of red/yellow carotenoid pigments in the skin [14-18]. Indeed, this skin color is a reliable biomarker of fruit and vegetable and carotenoid intake [19-22]. When asked to choose the skin color that looks healthiest in photographs of themselves or others, participants consistently choose a skin color that represents a higher fruit and vegetable consumption [14,23-25]. Likewise, when asked to choose the most attractive facial photograph, participants choose the one with increased carotenoid skin coloration over baseline coloration [26] and even over increased suntan coloration [27,28]. Some reports point to a limitation of the effect of carotenoid skin color. Appleton et al [29] found that carotenoid color did not affect attractiveness when pose and expression were unconstrained, and Tan et al [24] found that while a subtle level of carotenoid color increased attractiveness a large amount was deemed unattractive in a Malaysian Chinese population.

In terms of using the effect of fruit and vegetable consumption on skin color to motivate increased fruit and vegetable consumption, limited trials have been conducted. Whitehead et al [30] found that those shown a personalized demonstration of the potential skin color improvements arising from increased fruit and vegetable consumption reported an increase in fruit and vegetable consumption at a 10-week follow-up.

The present study aimed to assess the extent to which the effects of fruit and vegetable consumption on skin color are known within the UK. After extensive media coverage, we expected that the effect of fruit and vegetable consumption on skin color would be known by a proportion of the population, but the demographics of those familiar with the effect are unclear.

Secondly, we aimed to assess the extent to which UK residents are motivated to eat fruit and vegetables by a simple demonstration of the effects of fruit and vegetable consumption on skin color. Given the previous studies, we expected that a majority of individuals would express positive motivation following the online demonstration.

Specific subsections of society may be more receptive to appearance-based incentives than others. Within the US, Hayes and Ross [31] found younger participants to be more concerned about their appearance than older participants. Women were also more concerned about their appearance than men [31]. Across all demographic groups, the appearance was a powerful motivation for healthy eating [31]. By analyzing questionnaire data from 236 college-age women, Chung et al [32] found appearance (particularly weight maintenance) to be an essential factor in women’s decisions to eat fruit and vegetables. It was, therefore, predicted that younger and female participants would show the greatest motivation to increase fruit and vegetable consumption following the online demonstration of appearance changes related to diet. While it is clear that low socioeconomic status is predictive of reduced fruit and vegetable consumption [11,12], it is unclear whether socioeconomic status relates to the dietary motivation from skin color change.

## Methods

### Participant Enrollment

Ethical approval was obtained from the University of St Andrews School of Psychology Ethics Committee (PS13092). The study was completed with 802 participants recruited via Prolific, an online UK-based recruitment platform for surveys and behavioral experiments [33].

The Prolific company supplies sociodemographic information for their panelists. Prolific asks recruits to answer the following question: “Think of a ladder (image of a ladder with 10 rungs) as representing where people stand in society. At the top of the ladder are the people who are best off—those who have the most money, most education, and the best jobs. At the bottom are the people who are worst off—who have the least money, least education and the worst jobs or no job. The higher up you are on this ladder, the closer you are to people at the very top, and the lower you are, the closer you are to the bottom. Where would you put yourself on the ladder? Choose the number (1-10) whose position best represents where you would be on this ladder.”

Participants were recruited in four waves: (1) 202 18-26 year-olds who rated themselves as belonging to the lower half of self-perceived socioeconomic status (rating of 1-5 on the 10-point visual analog ladder scale), (2) 199 18-26 year-olds who rated themselves as belonging to the upper half of self-perceived socioeconomic status (6-10 rating on the 10 point scale), (3) 200 40-60 year-olds who rated themselves as belonging to the lower half of self-perceived socioeconomic status, and (4) 201 40-60 year-olds who rated themselves as belonging to the upper half of self-perceived socioeconomic status.

Participants were paid £0.6 for taking part (at an average rate of £9 per hour, 80% above stipulated minimum rate), with the questionnaire taking approximately 4 minutes to complete.

### Image Transforms

**Figure 1:**
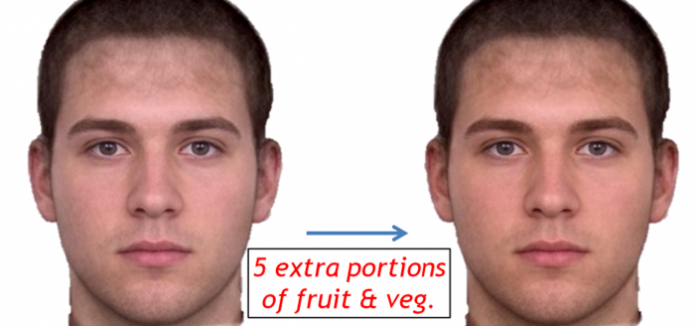
The image shown to participants before answering questions about the skin color benefits of fruit and vegetable consumption.

Participants were shown before and after images of a male face (Figure 1). The left-hand image was a composite image made by combining 13 images of men who had consented to their image being published. This image was then graphically changed to represent the skin color changes that occur following the consumption of 5 portions of fruit and vegetables for 6 weeks. Image transformation to simulate the effect of increasing dietary fruit and vegetables followed methods outlined previously [14,23,24,27]. This single image pair was used to make the demonstration as simple as possible.

The color change in the presented image pair was measured by selecting a large patch of the image of facial skin between the eyes and neck and analyzing the average color values of the pixels within the image patch. The right-hand image in Figure 1 was darker, redder, and yellower than the left image. The color difference between right and left images in CIELab color space was L*a*b*=-1.5, 2.0, 4.1. The displayed color difference is equivalent to the skin color change (L*a*b*=-1.8, 1.2, 3.8) after 4 weeks of a 500 mL/day dietary smoothie supplement of 6 extra daily portions of fruit and vegetables [15]. The color difference we use is one-quarter of the difference used in other studies [27,34].

### Procedure

Participants were first shown an image (Figure 1) showing the effect of fruit and vegetable consumption on skin color benefits. Participants were then given a questionnaire asking demographics (gender, age, ethnicity) and how many portions of fruit and vegetables they eat per day (0, 1, 2, 3, 4, 5, 6, 7, 8, 9, 10, 11 or more). They were also asked whether they were aware that diet affected skin color appearance and health. “Before seeing this display, were you aware that eating fruit and vegetables (a) can impart a golden glow to your skin color? (b) may help reduce the risk of the two main killer diseases in the UK—heart disease and some cancers?”. Answer options were: no, somewhat, yes (rescored as 0, 1, 2).

One question assessed the motivational effect of the display: “Has seeing the effect of diet on skin color in this exhibit made you want to eat less or more fruit and vegetables?”. Answer options were: a lot less, a little less, no change, a little more, a lot more.

Two further questions assessed the relative motivation of participants: “How much would the following make you want to eat more fruit and vegetables: (a) getting a skin color that looks healthy and attractive (within 20 days), (b) reducing your chance of heart disease and some forms of cancer?” Answer options were: none at all, a little, a moderate amount, a lot, a great deal.

### Analysis

Non-parametric statistics were used for ordinal data (awareness and motivation), and fruit and vegetable consumption as skew departed from normality. Mann-Whitney U tests were used to compare 2 groups, Kruskal-Wallis, to compare more than 2 groups and Wilcoxon Signed Rank test T to explore choice across a single group. Three of the 802 (<1%) participants reported consuming 11 or more portions of fruit and vegetables per day. Therefore, consumption data was approximated as an interval scale ranging from 0 to 11.

### Missing Data

For each variable, the analysis was restricted to participants with data either through their registration with Prolific (younger 18-26 vs older 40-60 age bracket, socioeconomic status) or through our questionnaire (age at the time of testing, gender, ethnicity). Data were missing for 36 participants (5%) for age at the time of testing, 20 (3%) for ethnicity, and 12 (1%) for socioeconomic status. Descriptions of the study population in terms of gender, age, and ethnicity are based on answers to our questionnaire. No attempt was made to compensate for missing data.

## Results

### Participant Demographics

#### Gender

Participants included 489 females (61%) and 311 males (39%). Two individuals reported their gender as “Other.”

#### Age

In our questionnaire, 381 of the 401 younger participants (in the age bracket 18-26) reported age at the time of testing age (mean 21.57, SD 2.09, range 18-30 years). Of the 401 older participants in the 40-60 age bracket, 385 reported age at the time of testing (mean 48.7, SD 5.83, range 38-61 years).

#### Socioeconomic Status

We observed 790 participants (1% missing data) who had Prolific categories of self-perceived socioeconomic status scale ranging from 1 (the lowest) to 10 (the highest), mean rating=5.32 (SD 1.67). The frequency of self-report rating was 12 (1%), 32 (4%), 72 (9%), 123 (20%), 157 (24%), 190 (19%), 148 (6%), 47 (1%), 4 (1%), and 5 (1%) across the 10 status levels (a distribution with acceptable skew –0.27 and kurtosis=0.17).

#### Ethnicity

We observed that 782 participants (2% missing data) chose an image that best represented their ethnic background from four images representing Black (African), White (Caucasian), East Asian, and West Asian (Indian/Pakistani). Of these, 182 participants (23%) identified themselves as having African ethnicity, 87 participants (11%) as Asian, 454 participants (57%) as Caucasian, 23 participants (3%) as West Asian, 36 participants (5%) as having more than one ethnicity, and 18 participants (2%) reported an ethnicity not shown by our questionnaire stimuli.

### Fruit and Vegetable Consumption

The mean level of fruit and vegetable consumption across the entire sample was mean 3.38 (SD 1.76) (skew 0.40 and kurtosis=0.10) portions per day. Young adults reported eating less than older adults (3.13, SD 1.72 vs 3.63, SD 1.76 portions per day; younger adults mean rank=363.9, N=399; older adults mean rank=436.9, N=401; Mann-Whitney U=94,601, *P*<.001, r=.161). Low socioeconomic status participants reported eating less than those of high socioeconomic status (3.15, SD 1.70 vs 3.62, SD 1.78 portions per day; low status mean rank=363.45, N=395; high status mean rank=425.71, N=393; U=89,884, *P*<.001, r=.139). Men reported eating less than women (3.11, SD 1.60 vs 3.55, SD 1.83 portions per day; men mean rank=367.25, N=310; women mean rank=419.99, N=488; U=85,638, *P*<.001, r=.114). Consumption of fruit and vegetables did not differ across three ethnic groups, Black, White, and Asian (N=721, Kruskal-Wallis Test=1.884, *df*=2, *P*=.39).

### Awareness of Appearance Benefits

We had 487 participants (61%) report they were unaware of the association between diet and skin color; 219 (27%) were somewhat aware, and 95 (12%) were aware. In contrast, 66 (8%) participants were unaware of the health risks (eg, cancer, heart disease) associated with a diet low in fruit and vegetables. Younger participants were more aware of the fruit and vegetable effect on skin color than older participants (younger adults mean rank=417.67, N=400; older adults mean rank=384.37, N=401; U=73,531, *P*=.02, r=–.081). Participants who reported low socioeconomic status were equally aware of the effect as participants reporting high socioeconomic status (low socioeconomic status mean rank=397.13, N=396; high socioeconomic status mean rank=392.86, N=393; U=76,972, *P*=.76, r=–.01). Women were more aware of the fruit and vegetable effect on skin color than men (women mean rank=416.67, N=489, men mean rank=373.71, N=310; U=83,946, *P*=.003, r=.104). Awareness of the fruit and vegetable effect on skin color differed across three ethnic groups, Black, White, and Asian (N=722, Kruskal-Wallis Test=7.915, *df*=2, *P*=.02). White participants showed the least awareness (Black mean rank=382.71, Asian mean rank=392.55, White mean rank=347.02).

### Motivation Change from Seeing an Effect on Skin Color

We observed 457 participants (57%) reporting that the simple demonstration of the dietary impact on skin color positively motivated them to eat more fruit and vegetables.

#### Age

**Figure 2:**
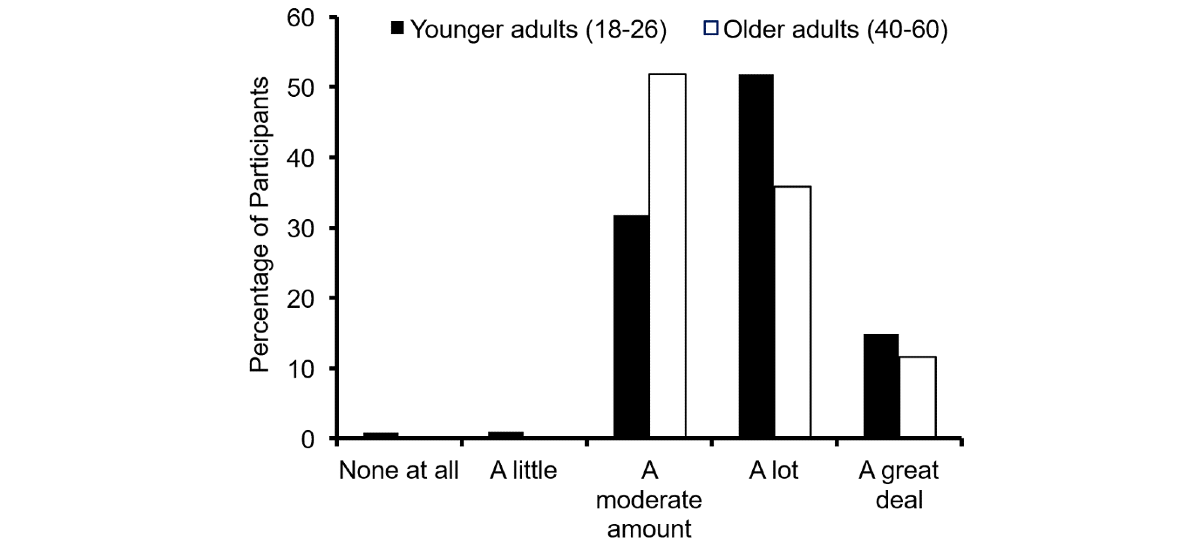
Effect of age on appearance motivation. Change in motivation to eat fruit and vegetables as a result of seeing the demonstration images for two age groups. Younger adults were more positively motivated than older participants.

Comparing younger (18-26 years old) and older participants (40-60 years old) showed that the younger group was more likely to endorse the view that the appearance demonstration motivated diet change than the older group (Figure 2, younger participants mean rank=435.79, N=400; older participants mean rank=366.30, N=401; U=66,284, *P*<.001, r=–.164).

#### Gender

Participant gender had no significant effect on the motivation for diet change from the demonstration of the effects of fruit and vegetables on skin color (women mean rank=408.37, N=489, men mean rank=386.80, N=310; U=79,886, *P*=.16, r=.05). The appearance demonstration was thus equally motivating to women and men.

#### Socioeconomic Status

Comparing low (self-reporting levels 1-5) and high socioeconomic status (self-reporting levels 6-10) participants showed that the two groups were equally likely to endorse the view that the appearance demonstration was motivating to change diet (mean rank low=396.12, N=396; mean rank high=393.88, N=393; U=77,372, *P*=.88, r=–.005). Hence, the appearance motivation was equal across socioeconomic status.

#### Ethnicity

The distribution of answers (to the question of whether the demonstration of skin color change motivated dietary change) across three ethnic groups, Black, White and Asian, was not significantly different (N=721, Kruskal-Wallis Test=2.999, *df*=2, *P*=.22). Comparing the median score of participants answering this question to a median of zero, the score expected by random choice, each ethnic group showed a significant bias to answer the question affirmatively (Black participants: One-Sample Wilcoxon Signed Rank test T=5,899.5, N=182, asymptotic two-tailed significance *P*<.001; Asian participants: T=1,573.5, N=87, *P*<.001; White participants: T=31,311, N=452, *P*<.001). Hence Black, White and Asian groups found the color demonstration motivating (Figure 1).

### Appearance vs Health Motivation for Dietary Fruit and Vegetables

**Figure 3:**
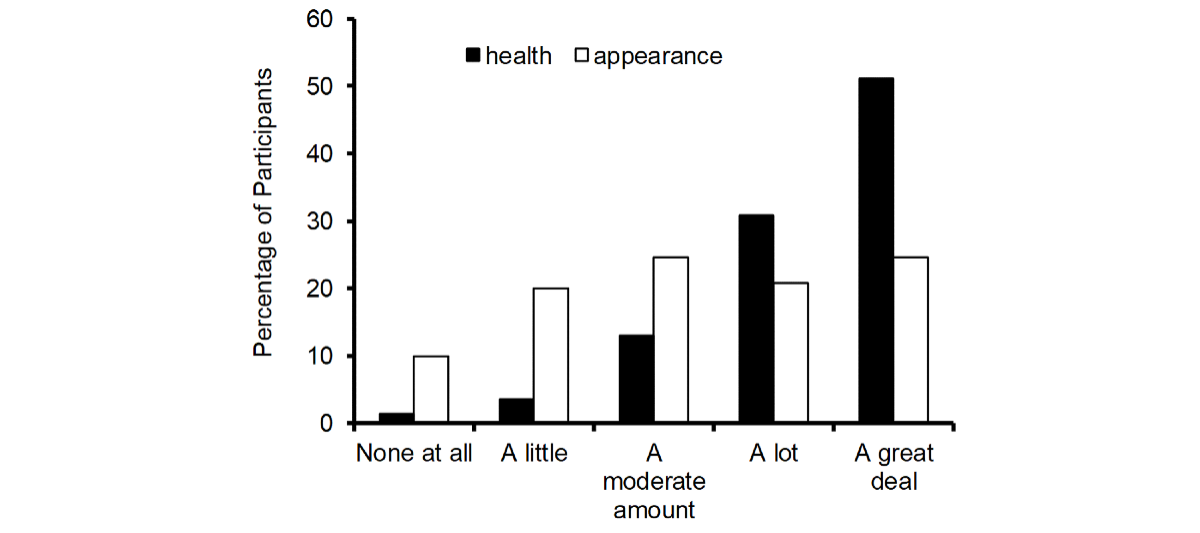
Relative motivation of health and appearance for the consumption of fruit and vegetables.

The vast majority (n=720, 90%) of participants answered that skin color appearance would motivate them to eat more fruit and vegetables. By contrast, virtually all participants (n=792, 99%) were motivated to a greater or lesser extent by the health benefits of fruit and vegetables in reducing the chance of heart disease and cancer (Figure 3). The response categories were recoded as a score (0-4) to compare the relative importance of health and appearance motivation. The mean numerical score for health motivation was 3.27, while the mean score for appearance was 2.30 (Wilcoxon Signed-Rank test T=118,103; N=801, *P*<.001, r=.618).

#### Gender

*A*n appearance/health contrast score was computed for each participant (the 0-4 score of the importance of appearance minus the 0-4 score of the importance of health for consuming fruit and vegetables) to compare health and appearance motivation. Comparing contrast scores for women and men showed that the relative importance of appearance to health in motivating diet change was higher for women (mean rank=416.29, N=489) than men (mean rank=374.3, N=310; U=83,763, *P*=.01, r=.09).

#### Age

Younger adults (mean rank=442.96, N=400) found the appearance benefits more motivating relative to the health benefits (mean rank=359.15, N=401, U=63,416, *P*<.001, r=.19).

#### Socioeconomic Status

There was no effect of socioeconomic status on the relative motivation of health and appearance (mean rank high=392.52, N=393; mean rank low=397.46, N=396; U=78,839, *P*=.75, r=–.011).

#### Knowledge of the Effect of Fruit and Vegetables on Skin Color

The importance of appearance relative to health in motivating diet change was higher for those participants who already knew that diet alters skin color (mean rank=445.79, N=314) than for those not aware of the effect (mean rank=372.12, N=487, U=90,522, *P*<.001, r=.161).

## Discussion

### Principal Findings

The primary findings of the study were (a) the limited percentage of people (40%) with knowledge of the dietary–skin color association and (b) that many people (57%) found the simple demonstration of the dietary impact on skin color positively motivating to eat more fruit and vegetables, while very few (1%) reported the demonstration was demotivating. The combination of these two findings points to the benefits that can be accrued through publicizing and demonstrating the dietary effect on skin color. Media coverage in the UK has been extensive (reaching millions of TV viewers), but there is still a substantial proportion of the population that reports not knowing the dietary effects. Of the 801 participants, 487 (61%) were unaware of the effects of fruit and vegetable consumption on skin color. Of these, 248 (51%) stated they were motivated to eat more fruit and vegetables as a result of seeing the demonstration. Hence increasing public awareness of the dietary effects on the skin could provide additional motivation for a healthy diet for 31% of the sample population (% of those unaware multiplied by % of those motivated). Extrapolating this to the UK adult population of approximately 50 million, this amounts to 16.5 million people.

### Age and Socioeconomic Status

Low socioeconomic status and young adult age are established independent categories predicting low fruit and vegetable consumption [10,11]. In the current study, it was again evident that the younger adults and those reporting low socioeconomic status were reporting lower fruit and vegetable consumption. It is noteworthy that the motivational effects of appearance were most prominent in younger adults. By contrast, we were unable to detect any influence of socioeconomic status on appearance motivation. Hence, it is reasonable to expect that promotion of appearance benefit would not widen health inequalities, and drawing attention to the color benefits of a healthy diet would have an impact on a younger population independent of socioeconomic status, thereby increasing the chance of their life-long adoption of an improved diet.

### Gender

We expected women to be more motivated by appearance benefits than men. Comparing reactions to the visual demonstration, we found no significant impact of gender. For both men and women in the present study, motivation for a diet high in fruit and vegetables was more significant for the benefits to health than the motivation for an improved skin color appearance. Nonetheless, motivation from benefits to appearance relative to benefits to health was higher in women than in men.

### Fruit and Vegetable Consumption

The average consumption of fruit and vegetables reported by the entire sample here was 3 portions per day (significantly less than the 5 a day recommended by the British National Healthcare System). Hence publicity of the appearance benefits of a proper diet rich in fruit and vegetables could augment motivation for a healthy diet. It is relevant here that the majority of our participants (57%) reported that the appearance benefits were positively motivating. Indeed, appearance has proved valuable in encouraging other aspects of healthy behavior (eg, sun protection) [35].

### Ethnicity Effects

The participants in this study reported a variety of ethnic backgrounds (23% Black, 11% Asian, and 57% White). The effect of carotenoids is likely to be less apparent for individuals with darkly pigmented skin. Nonetheless, carotenoid effects on skin color are apparent across different ethnicities. Coetzee & Perrett [36] found that carotenoid supplementation produced an increase in skin yellowness in sun-protected skin regions of African participants. Likewise, a diet rich in fruit and vegetables increases skin yellowness in Asian participants [15,24] as it does in White participants [14,16,26].

While the skin color effects of diet are detectable in a range of ethnic groups, different cultures may vary in their perception of whether the skin color change is desirable. Black South African participants were found to perceive increased skin yellowness positively [23]. However, a recent report indicates that Asian participants from Malaysia find only a slight increase in carotenoid skin color more attractive than the skin color baseline [24]. A further report concludes that mainland Chinese participants do not find increased yellow skin pigmentation attractive [37]. Nonetheless, whatever the influence of culture on color preferences, the current study found that Asian, Black, and White participants from the UK were equally impressed by the impact of diet on skin color.

### Limitations

We detected several factors associated with attitudes to the appearance benefit arising from a diet high in fruit and vegetables. Socioeconomic status did not influence appearance motivation. Our measures of socioeconomic status and dietary intake were self-reported, and portions of fruit and vegetables were not defined (eg, as 80 g). More objective measures and greater sampling of those with low and high socioeconomic status individuals are needed to be confident in null effects. We have not assessed residential area deprivation [11] or sense of personal relative deprivation [38], both predictors of diet and self-reported health.

We illustrated the dietary effect with a single Caucasian male face. The demonstration may be more effective if displayed with the same gender, age, and ethnicity as the participant or better still with an image of the participant [30]. The demonstration was made early in the survey, which may have led to participants increasing their reported fruit and vegetable consumption. The dietary effect on skin color is not unique since other aspects of lifestyle (eg, fitness and reduced body fat) have similar impacts on skin color appearance [39].

We measured the effects of appearance on self-reported motivation to consume more fruit and vegetables. We acknowledge that motivation is necessary but not sufficient for actual dietary change [40]. The impact of appearance on real dietary change is likely to be smaller than the impact on motivation to change. Translating motivation into an actual increase in fruit and vegetable consumption remains an issue that is affected by a person’s perception that they can control their behavior [14,40,41]. Nonetheless, any change in motivation to consume more fruit and vegetables is an essential step in the right direction of a healthy lifestyle.

## References

[1] Aune D, Giovannucci E, Boffetta P, Fadnes LT, Keum N, Norat T, Greenwood DC, Riboli E, Vatten LJ, Tonstad S (2017). Fruit and vegetable intake and the risk of cardiovascular disease, total cancer and all-cause mortality-a systematic review and dose-response meta-analysis of prospective studies. Int J Epidemiol.

[2] Wang X, Ouyang Y, Liu J, Zhu M, Zhao G, Bao W, Hu FB (2014). Fruit and vegetable consumption and mortality from all causes, cardiovascular disease, and cancer: systematic review and dose-response meta-analysis of prospective cohort studies. BMJ.

[3] Hu D, Huang J, Wang Y, Zhang D, Qu Y (2014). Fruits and Vegetables Consumption and Risk of Stroke. Stroke.

[4] Li M, Fan Y, Zhang X, Hou W, Tang Z (2014). Fruit and vegetable intake and risk of type 2 diabetes mellitus: meta-analysis of prospective cohort studies. BMJ Open.

[5] Du H, Li L, Bennett D, Guo Y, Turnbull I, Yang L, Bragg F, Bian Z, Chen Y, Chen J, Millwood IY, Sansome S, Ma L, Huang Y, Zhang N, Zheng X, Sun Q, Key TJ, Collins R, Peto R, Chen Z, China Kadoorie Biobank study (2017). Fresh fruit consumption in relation to incident diabetes and diabetic vascular complications: A 7-y prospective study of 0.5 million Chinese adults. PLoS Med.

[6] Yip Cynthia Sau Chun, Chan Wendy, Fielding Richard (2019). The Associations of Fruit and Vegetable Intakes with Burden of Diseases: A Systematic Review of Meta-Analyses. J Acad Nutr Diet.

[7] GBD 2017 Diet Collaborators (2019). Health effects of dietary risks in 195 countries, 1990-2017: a systematic analysis for the Global Burden of Disease Study 2017. Lancet.

[8] The Economist Intelligence Unit (2015). Financing the future: Choices and challenges in global health.

[9] (2017). Health Survey for England Summary of key findings.

[10] (2019). The National Diet and Nutrition Survey, Public Health England.

[11] Shohaimi S, Welch A, Bingham S, Luben R, Day N, Wareham N, Khaw K (2004). Residential area deprivation predicts fruit and vegetable consumption independently of individual educational level and occupational social class: a cross sectional population study in the Norfolk cohort of the European Prospective Investigation into Cancer (EPIC-Norfolk). J Epidemiol Community Health.

[12] Lakshman R, McConville A, How S, Flowers J, Wareham N, Cosford P (2011). Association between area-level socioeconomic deprivation and a cluster of behavioural risk factors: cross-sectional, population-based study. J Public Health (Oxf).

[13] Michie S, Abraham C, Whittington C, McAteer J, Gupta S (2009). Effective techniques in healthy eating and physical activity interventions: a meta-regression. Health Psychol.

[14] Whitehead RD, Re D, Xiao D, Ozakinci G, Perrett DI (2012). You are what you eat: within-subject increases in fruit and vegetable consumption confer beneficial skin-color changes. PLoS One.

[15] Tan KW, Graf BA, Mitra SR, Stephen ID (2015). Daily Consumption of a Fruit and Vegetable Smoothie Alters Facial Skin Color. PLoS One.

[16] Pezdirc K, Hutchesson MJ, Whitehead R, Ozakinci G, Perrett D, Collins CE (2015). Fruit, Vegetable and Dietary Carotenoid Intakes Explain Variation in Skin-Color in Young Caucasian Women: A Cross-Sectional Study. Nutrients.

[17] Pezdirc K, Hutchesson MJ, Williams RL, Rollo ME, Burrows TL, Wood LG, Oldmeadow C, Collins CE (2016). Consuming High-Carotenoid Fruit and Vegetables Influences Skin Yellowness and Plasma Carotenoids in Young Women: A Single-Blind Randomized Crossover Trial. J Acad Nutr Diet.

[18] Bixley G, Clark K, James A (2018). Skin colour predicts fruit and vegetable intake in young Caucasian men: A cross-sectional study. Journal of Nutrition & Intermediary Metabolism.

[19] Mayne ST, Cartmel B, Scarmo S, Lin H, Leffell DJ, Welch E, Ermakov I, Bhosale P, Bernstein PS, Gellermann W (2010). Noninvasive assessment of dermal carotenoids as a biomarker of fruit and vegetable intake. Am J Clin Nutr.

[20] Scarmo S, Henebery K, Peracchio H, Cartmel B, Lin H, Ermakov IV, Gellermann W, Bernstein PS, Duffy VB, Mayne ST (2012). Skin carotenoid status measured by resonance Raman spectroscopy as a biomarker of fruit and vegetable intake in preschool children. Eur J Clin Nutr.

[21] Darvin M, Patzelt A, Knorr F, Blume-Peytavi U, Sterry W, Lademann J (2008). One-year study on the variation of carotenoid antioxidant substances in living human skin: influence of dietary supplementation and stress factors. J Biomed Opt.

[22] Ermakov IV, Ermakova M, Sharifzadeh M, Gorusupudi A, Farnsworth K, Bernstein PS, Stookey J, Evans J, Arana T, Tao-Lew L, Isman C, Clayton A, Obana A, Whigham L, Redelfs AH, Jahns L, Gellermann W (2018). Optical assessment of skin carotenoid status as a biomarker of vegetable and fruit intake. Arch Biochem Biophys.

[23] Stephen ID, Coetzee V, Perrett DI (2011). Carotenoid and melanin pigment coloration affect perceived human health. Evolution and Human Behavior.

[24] Tan KW, Graf BA, Mitra SR, Stephen ID (2017). Impact of fresh fruit smoothie consumption on apparent health of Asian faces. Evolution and Human Behavior.

[25] Pezdirc K, Rollo ME, Whitehead R, Hutchesson MJ, Ozakinci G, Perrett D, Collins CE (2017). Perceptions of carotenoid and melanin colouration in faces among young Australian adults. Aust J Psychol.

[26] Foo Y, Rhodes G, Simmons L (2017). The carotenoid beta-carotene enhances facial color, attractiveness and perceived health, but not actual health, in humans. Behav Ecol.

[27] Lefevre CE, Perrett DI (2015). Fruit over sunbed: carotenoid skin colouration is found more attractive than melanin colouration. Q J Exp Psychol (Hove).

[28] Whitehead RD, Ozakinci G, Perrett DI (2012). Attractive skin coloration: harnessing sexual selection to improve diet and health. Evol Psychol.

[29] Appleton KM, McGrath AJ, McKinley MC, Draffin CR, Hamill LL, Young IS, Woodside JV (2018). The value of facial attractiveness for encouraging fruit and vegetable consumption: analyses from a randomized controlled trial. BMC Public Health.

[30] Whitehead RD, Ozakinci G, Perrett DI (2014). A randomized controlled trial of an appearance-based dietary intervention. Health Psychol.

[31] Hayes D, Ross CE (1987). Concern with appearance, health beliefs, and eating habits. J Health Soc Behav.

[32] Chung S, Hoerr S, Levine R, Coleman G (2006). Processes underlying young women's decisions to eat fruits and vegetables. J Hum Nutr Diet.

[33] (2020). Prolific.

[34] Lefevre CE, Ewbank MP, Calder AJ, von dem Hagen E, Perrett DI (2013). It is all in the face: carotenoid skin coloration loses attractiveness outside the face. Biol Lett.

[35] Williams AL, Grogan S, Clark-Carter D, Buckley E (2013). Appearance-based interventions to reduce ultraviolet exposure and/or increase sun protection intentions and behaviours: a systematic review and meta-analyses. Br J Health Psychol.

[36] Coetzee V, Perrett DI (2014). Effect of beta-carotene supplementation on African skin. J Biomed Opt.

[37] Han C, Wang H, Hahn AC, Fisher CI, Kandrik M, Fasolt V, Morrison DK, Lee AJ, Holzleitner IJ, DeBruine LM, Jones BC (2018). Cultural differences in preferences for facial coloration. Evolution and Human Behavior.

[38] Callan MJ, Kim H, Matthews WJ (2015). Predicting self-rated mental and physical health: the contributions of subjective socioeconomic status and personal relative deprivation. Front Psychol.

[39] Perrett DI, Talamas SN, Cairns P, Henderson AJ (2020). Skin Color Cues to Human Health: Carotenoids, Aerobic Fitness, and Body Fat. Front Psychol.

[40] Sniehotta FF, Scholz U, Schwarzer R (2005). Bridging the intention–behaviour gap: Planning, self-efficacy, and action control in the adoption and maintenance of physical exercise. Psychology & Health.

[41] Menozzi D, Sogari G, Mora C (2015). Explaining Vegetable Consumption among Young Adults: An Application of the Theory of Planned Behaviour. Nutrients.

